# Risk Factors for Retinal Ganglion Cell Distress in Glaucoma and Neuroprotective Potential Intervention

**DOI:** 10.3390/ijms22157994

**Published:** 2021-07-27

**Authors:** Stefania Vernazza, Francesco Oddone, Sara Tirendi, Anna Maria Bassi

**Affiliations:** 1 Department of Experimental Medicine (DIMES), University of Genoa, 16126 Genoa, Italy; tirendisara@gmail.com (S.T.); Anna.Maria.Bassi@unige.it (A.M.B.); 2 IRCCS-Fondazione Bietti, 00198 Rome, Italy; francesco.oddone@fondazionebietti.it; 3 Inter-University Center for the Promotion of the 3Rs Principles in Teaching & Research (Centro 3R), 56122 Pisa, Italy

**Keywords:** neuroprotection, glaucoma, retinal ganglion cells, neuroinflammation, degeneration

## Abstract

Retinal ganglion cells (RGCs) are a population of neurons of the central nervous system (CNS) extending with their soma to the inner retina and with their axons to the optic nerve. Glaucoma represents a group of neurodegenerative diseases where the slow progressive death of RGCs results in a permanent loss of vision. To date, although Intra Ocular Pressure (IOP) is considered the main therapeutic target, the precise mechanisms by which RGCs die in glaucoma have not yet been clarified. In fact, Primary Open Angle Glaucoma (POAG), which is the most common glaucoma form, also occurs without elevated IOP. This present review provides a summary of some pathological conditions, i.e., axonal transport blockade, glutamate excitotoxicity and changes in pro-inflammatory cytokines along the RGC projection, all involved in the glaucoma cascade. Moreover, neuro-protective therapeutic approaches, which aim to improve RGC degeneration, have also been taken into consideration.

## 1. Introduction

Glaucoma represents a group of neurodegenerative diseases characterized by optic nerve damage and the slow progressive death of retinal ganglion cells (RGCs). Indeed, glaucoma is regarded as the second cause of irreversible blindness worldwide and it is estimated that its incidence will increase to more than 112 million cases in the future [1,2,3].

In connection with an increased susceptibility to glaucoma and the progression of such a disease, several risk factors, including age, Intra Ocular Pressure (IOP), race, severe myopia, genetic background, vascular dysregulation and central corneal thickness, have been identified [4,5]. Such a multitude of risk factors can be explained by glaucoma etiological complexity, as well as by the several glaucoma forms in which pressure does not increase [6]. Indeed, although IOP has been recognized as one of the main glaucoma risk factors due to it being responsible for both mechanical axonal damage and nutrient interruption [5,7], there is a particular glaucoma form, corresponding to 20–25% of glaucomatous optic neuropathy, characterized by IOP within a normal range, i.e., Normal Tension Glaucoma (NTG), which, however, leads to progressive blindness. Moreover, not all people who have elevated IOP suffer from glaucoma [8,9,10]. 

From an anatomical point of view all glaucoma forms are classified in two types, i.e., the open angle and closed-angle glaucoma, according to the geometry of iridocorneal angle, the point where the iris and the cornea meet [6]. However, Primary Open Angle Glaucoma (POAG) is the most common glaucoma type, accounting for over 70% of cases. 

Currently, clinical treatments for all glaucoma types aim for lowering IOP through topical hypotensive drugs or surgery. However, these approaches are not sufficiently successful for many patients who continue to lose their vision [11]. Therefore, it would seem evident that RGC death is also driven by different converging molecular pathways, engaged in additional damage more or less closely connected with IOP elevation, which are able to trigger or exacerbate the glaucomatous cascade. The purpose of this present review is to summarize the most recent evidence about some of the possible upstream causes which are responsible for RGC death, as well as neuroprotective strategies to prevent or at least to slow down progression of the retinal distress.

## 2. Retinal Ganglion Cells

RGCs, in addition to cone photoreceptors and horizontal, amacrine and bipolar cells, as well as Müller glia, all belong to the neural cells of the vertebrate retina [12]. Although they share a common multi-potent retinal progenitor cell (RPC), RGCs are the first to be generated. 

During the embryonic phase, by way of Sonic Hedgehog (SHH), RGCs exert a control over different downstream molecular events, including the laminar organization of the retina, the size of successive cell populations, the development of optic stalk neuro-epithelial cells and their morphogenesis [12,13,14,15]. In the advanced stages of development, the RGC axons of the optic nerve, which form the retinal nerve fiber layer, are closely involved with the migrating astrocytes. The Roundabout (Robo) single-pass type I trans-membrane family receptors are key regulators of commissural axon guidance, providing and controlling the complex neuronal networks [16]. In fact, by way of *Robo 1* and *Robo 2*, RGCs provide directional information to the astrocytes, i.e., the centrifugal trajectory, which is useful for their polarization and for the colonization of the peripheral retina [14,17]. Moreover, RGCs and their axons are responsible for transmitting all the visual information from the eye to the brain (i.e., lateral geniculate nucleus of the thalamus and superior colliculus of the midbrain) through the optic nerve [18]. 

Amongst all the neuron types, RGCs, due to their morphological features and their high energy requirements, are particularly vulnerable to mitochondrial dysfunction. In fact, a lack of saltatory conduction, due to unmyelinated axons of RGCs within the retina, leads to a higher requirement of more mitochondria in order to respond to the high metabolic demand [19]. Therefore, to provide RGCs with the correct energy contribution, the mitochondria integrity, in terms of their biogenesis, dynamics, transport and degradation (e.g., mitophagy), is required. The presence of mitochondria deficits, triggered by mutations in genes encoding essential mitochondrial functions, as in the case of aging, results in an inadequate energy supply which is involved in RGC degeneration [20,21]. 

Several diseases can occur as a result of either RGC axon dysfunction or their degeneration, including glaucomatous optic neuropathy, inflammatory optic neuropathy, ischemic optic neuropathy, traumatic optic neuropathy, hereditary optic neuropathy and toxic/nutritional optic neuropathy [22]. 

In particular, the neuropathy characteristics of different glaucoma types include permanent visual deficits that begin with peripheral vision loss, degenerating towards the so-called “tunnel vision” and, eventually, the loss of sight [23]. 

**Figure 1:**
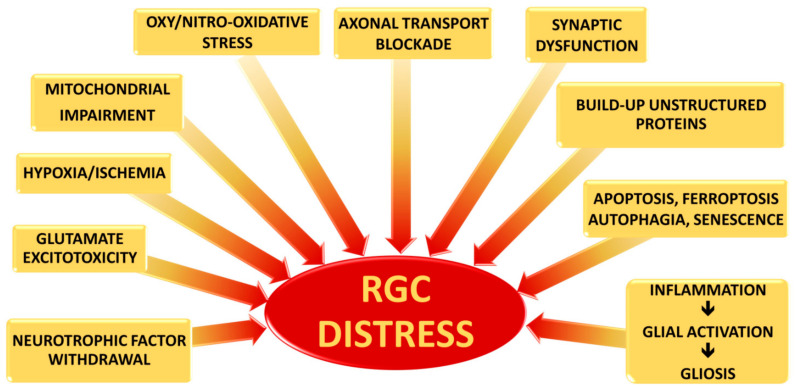
Risk factors contributing to RGC distress in glaucoma: the mechanisms implicated in RGC damage include deficiency in neurotrophic elements, glutamate excitotoxicity, hypoxia and ischemia, impairment of mitochondria functions, oxidative and nitrosative stress, alterations in axonal transport and in synaptic signals, unstructured proteins, apoptosis and other death mechanisms which can lead to premature senescence, and inflammation cascade which induce the activation of glial cells with consequent gliosis.The pathways, triggered by each risk factor, often result to be strictly interrelated, contributing to amplifying RGC distress in an irreversible way.

Although the precise mechanisms by which RGCs die in glaucoma have not yet been clarified, three main causes of severe and extensive damage have been proposed: (i) axonal transport blockade; (ii) Glutamate excitotoxicity; and (iii) changes in pro-inflammatory cytokine along the RGC projection (Figure 1).

### 2.1. Axonal Transport Blockade

Undoubtedly, axonal transport is an essential process for neuron survival and, overall, for the maintenance of both the metabolic balance and intracellular neurotransmission. Moreover, it allows the neuron to respond effectively to trophic signals or stress insults. Such a process is distinguished in anterograde and retrograde axonal transport. The anterograde axonal transport, in addition to providing proteins and lipids from the soma to the distal synapses, also supplies local energy by way of a targeted mitochondria movement [24]. However, the retrograde transport is involved in the misfolded protein removal from the axon and in the intracellular transport of distal trophic signals to the soma in order to avoid toxic aggregate formation [25]. 

The axonal transport along the microtubule network, which can be divided into fast (0.5–4 µm/s) and slow (0.01–0.05 µm/s) transport according to the type of transported cargo [24,26], takes place thanks to the so-called molecular motors, i.e., the kinesin and dynein complexes. Although both require ATP to propel their cargo along the microtubule rails, kinesin is involved in the anterograde transport while dynein in the retrograde one [27].

It is now known that a defective axonal transport, related to the malfunctioning of one or more involved components (e.g., molecular motors, microtubules, cargo, or mitochondria), results in a predisposition to neurodegenerative diseases such as Alzheimer’s disease (AD) and other dementia, Parkinson’s disease (PD), Huntington’s disease, Charcot–Marie–Tooth peripheral neuropathy, amyotrophic lateral sclerosis and glaucoma [28,29].

Increasing evidence has highlighted that the neurodegeneration outcome can change according to the different involvement of defects borne by mutations in the motors that drive the anterograde and/or retrograde axon transport [25,30,31,32,33,34].

In particular, in glaucoma neurodegeneration, long-lasting harmful stimuli, such as low levels of oxygen or energy, the presence of neurotoxins, an increased pressure gradient across RGCs axonsand pro-inflammatory cytokines, initially promote an anterograde transport deficit and, successively, a retrograde one from the RGC body [7,20]. Although the presence of cytoskeleton and/or protein abnormalities, as well as metabolic deficits, are all clear signs of a pre-degenerative state, they do not necessarily affect the axonal function immediately [35]. However, the progressive axonal transport blockade, which is responsible for trophic signal deprivation (e.g., neurotrophic factors), plays an essential role in promoting RGC loss [36]. 

As known, neurotrophic factors (NTFs) are critical for the differentiation and maintenance of the nervous system, as well as for the support of neuronal cell survival. NTFs generally include: the neutrophin family, the glial cell-line derived neurotrophic factor (GDNF) family and the family represented by Ciliary Neurotrophic Factor (CNTF) [37,38].

As for neurotrophins (NTs), they are a family of polypeptides with multiple functions, both at peripheral and central nervous system level, ranging from neuronal survival and differentiation to the modulation of synaptic transmission and plasticity [39,40]. 

There are four NTs, namely Nerve Growth Factor (NGF) [41,42], Brain-Derived Neurotrophic Factor (BDNF) [43], NT-3 [44] and NT-4/5 [45], which are all characterized by homologies in terms of their sequence and structure [46]. These NTs bind two different classes of transmembrane receptors: the p75 NT receptor (p75^NTR^) and three members of Tropomyosin-Related Kinase (Trk) receptor tyrosine kinases, i.e., TrkA, TrkB and TrkC [47]. Therefore, NTs, through p75^NTR^, control both the neuron fate, via the NF-kB or Jun kinase pathways, and the motility of cone growth regulating Rho activity [48,49]. On the other hand, NTs, through Trk receptors, activate pro-survival and/or pro-growth signaling cascades, such as Ras/Raf/MEK/ERK and PI3-K/Akt/mTOR pathways [47,50].

Among NTs, BDNF seems to be particularly crucial for RGC survival because, in addition to being produced by the RGCs themselves and by retinal astrocytes, it also has a role in retrograde axonal transport (from the brain to the retina) [51,52]. Indeed, BDNF, once produced by the superior colliculus or by the lateral geniculate nucleus, binds to the TrkB receptor in order to reach the cell bodies by way of retrograde transport within the microsomal vesicles [53].

Previous studies of induced glaucoma animal models have shown that ocular hypertension is responsible for the obstruction of retrograde axonal transport at the optic nerve head to the point of affecting the distribution of both BDNF and TrkB along the RGCs, thus promoting RGC death [54,55]. In order to support these results, it has been shown that either multiple intravitreal injections of BDNF or intravitreal treatment with a BDNF-expressing adenoviral vector can actually temporarily slow down RGC loss [54,56,57].

However, RGC death, induced by ocular hypertension, can also be explained in another way. Indeed, in some cases, changes in both the mRNA and protein of NT receptors favor the activation of the p75-mediated pro-apoptotic signal, resulting in the alteration of the effects of NTs on RGC survival [58,59,60].

The GDNF family (i.e., GDNF, neurturin, artemin and persephin) is a distant member of the transforming growth factor-β (TGF-β) superfamily. Given its powerful neuroprotective role on various neurons, it has also been proposed as a target for treating several neurodegenerative diseases (e.g., AD and PD) [61,62,63].

In addition, it seems that GDNF acts on retinal degeneration both indirectly, stimulating the photoreceptor survival [41,42], and directly, after intravitreal injection of microspheres containing GDNF [64,65,66,67]. 

At a Müller glia level, a further interesting aspect of GDNF is its ability in enhancing the gene expression of the glutamate/aspartate transporter (GLAST), which is essential for RGC protection [68]. GLAST, together with Glutamate Transporter 1 (GLT-1) located in the bipolar cell terminals, and the Excitatory Amino Acids Carrier 1 (EAAC1) in retinal neurons including RGCs, are the only mechanisms for glutamate removal from the extracellular fluid in the retina. Moreover, GLAST is also important for glutathione synthesis, a major cellular antioxidant in the retina.

Although the excitotoxicity of glutamate in glaucoma pathogenesis is still controversial, in a mouse model, in which GLAST and EAAC1 were knocked-out, it has been shown that their absence promoted RGC death and glaucomatous damage, even without ocular hypertension [65]. 

Moreover, since CNTF belongs to the neuropoietic cytokine family, itis expressed by different cells in the retina and especially by Müller Glia. In addition to enhancing the survival of both photoreceptors and RGCs, CNTF is capable of stimulating axonal regeneration [69]. The specific binding of CNTF to the receptor complex, which includes the CNTF receptor-α (CNTFRα), gp130 and Leukemia Inhibitory Factor receptor (LIFR), activates Jak-STAT, MEK-ERK and PI3K/Akt signaling pathways [70,71]. 

The protective role of CNTF in eye-sight maintenance has been elucidated by aqueous humor analysis of POAG patients in which the severity of the disease correlated to the increased reduction in CNTF levels [72]. Moreover, studies on animal models have shown a progressive up-regulation of CNTF levels after optic nerve damage but only during the first weeks and not beyond [73,74]. 

### 2.2. Glutamate Excitotoxicity

Glutamate is the most abundant excitatory neurotransmitter in the mammalian CNS and is also involved in most of excitatory neurotransmissions when bound to its specific receptors.

Thus, in the CNS, glutamate is mainly located intracellularly even though, during synaptic transmission when its extracellular concentration increases, its levels in the synaptic cleft at resting conditions are kept low by the glutamate uptake/transporter system, which actively removes the residual glutamate [75,76]. 

Glutamate binds mainly to ligand-gated ionotropic glutamate receptors (iGluRs), which are classified into two groups: N-methyl D-aspartate (NMDA) receptors and non-NMDA receptors (i.e., AMPA and kainate receptors) [77,78]. These play fundamental roles in synaptic plasticity [79] and, in particular, NMDA receptors are also essential for neuron survival by activating the neuronal survival pathway [80,81]. At resting membrane potentials, glutamate cannot activate the NMDA receptors due to a control mechanism, consisting of magnesium ions (Mg^2+^), which blocks the intracellular influx of sodium (Na^+^) and calcium ions (Ca^2+^). However, this blockade by Mg^2+^, being voltage-dependent, is lost after neuron depolarization, enabling NMDA receptors to be activated by glutamate and also allowing the influx of sodium (Na^+^) and calcium ions (Ca^2+^), as well as the efflux of potassium ions (K^+^) [82]. Interestingly, on astrocyte and oligodendrocyte membranes, several receptors, belonging to the NMDA type, but with different subunit compositions, have been found. It would appear that their activation occurs without antecedent depolarization because they have not the blockade by Mg^2+^ [83,84]. 

An increase in extracellular glutamate concentration may be observed under several pathological conditions (e.g., ischemia/reperfusion injury, oxidative stress, inflammation and as a result of aging) due to a reduced activity of the glutamate uptake/transporter system [85,86,87,88]. The abundance of glutamate, in turn, results in nervous tissue damage which can alter physiological brain functions. Moreover, since TNFα increases the glutaminase expression on astrocytes, it is also responsible for increasing the glutamate amount [89]. 

A progressive glutamate accumulation in the extracellular space leads to glutamate excitotoxicity. Therefore, excitotoxicity injury refers to a condition in which the ionic homeostasis is lost due to an NMDA receptor overstimulation by glutamatergic neurotransmission. Since such damage leads to cell death, it has been proposed as a possible mechanism involved in neurodegenerative conditions, including AD and glaucoma [82,90,91]. However, increasing evidence indicates that an excess of glutamate is responsible for the hyperactivation of the glutamate receptors but the resulting massive Ca^2+^ influx into the post synaptic neurons is also due to low ATP levels, which prevent the proper functioning of the ion pumps [92]. Such intracellular accumulation of Ca^2+^ ions represents the trigger for activation of both the protein-kinases and other downstream Ca^2+^-dependent enzymes, which through a series of molecular modifications, such as cellular membrane damage, ROS release, mitochondrial dysfunction, lead to cell death [91,93]. 

Although the direct role of glutamate excitotoxicity in glaucoma pathogenesis remains circumstantial [94,95,96,97], its role has been demonstrated during both ischemic and hypoxia injuries [98,99]. Therefore, since both conditions have been found in glaucoma, it is conceivable that glutamate excitotoxicity could be involved in RGC death as a possible outcome of extensive damage rather than being the main cause [100]. 

Under prolonged hypoxic-ischemic conditions, the neural protective mechanisms are lost, leading to cell death and tissue damage. Therefore, under such conditions, neuronal degeneration can result from different detrimental mechanisms triggered by oxygen and substrate deprivation, including glutamate excitotoxicity, free oxygen radicals, and pro-inflammatory cytokines, causing a loss in the function of the blood retinal barrier (BRB) [100]. 

### 2.3. Changes in Pro-Inflammatory Cytokine along the RGC Projection

As known, inflammation is defined as an unavoidable cellular response to a prolonged period of noxious stress or tissue malfunction, characterized by a marked release in pro-inflammatory cytokines/chemokines and tissue damage. Although the para-inflammation condition refers to an adaptive-tissue response, necessary for maintaining tissue homeostasis and restoring its functionality, either an increase in its magnitude or its dysfunction leads to the loss of its physiological role, thus resulting in an inflammation state [101]. 

Increasing evidence suggests that oxidative stress and ocular inflammation are the main factors involved in the pathogenic mechanisms that lead to the neurodegeneration process found in NTG and high tension glaucoma (HTG) [102,103,104]. 

Under physiological conditions, structural and functional integrity of the retina is provided by a regular oxygen supply since it is one of the most metabolically-active tissues [105]. However, pathological conditions, such as central retinal artery occlusion and ischemic central retinal vein thrombosis, lead to retinal hypoxia-ischemia, which is involved in several diseases including glaucoma [106,107,108,109]. As above-described (see Section Glutamate Excitotoxicity), during retinal hypoxia-ischemia, a series of cellular alterations, such as the increase in the free oxygen radical production, the activation of the inflammatory pathway, the glutamate excitotoxicity and the destruction of the BRB, occur. 

In particular, the strong connection between the retinal ischemic episode and the increase in Reactive Oxygen Species (ROS) production is of great interest. In fact, when normal blood-flow is re-established, the ischemic injury gets even worse due to re-oxygenation, which represents an important mechanism of cellular damage and ROS production. In this regard, three phases of ROS generation have been distinguished: the first one is sustained by mitochondria in response to the inhibition of mitochondrial respiration, the second by xanthine oxidase activation, due to ATP depletion, and the third, by the calcium-dependent activation of Nicotinamide Adenine Dinucleotide Phosphate (NADPH) oxidase [110]. This massive increase in ROS stimulates the damaged cells to produce inflammatory cytokines, such as TNFα and interleukins (IL), as well as vascular endothelial growth factor (VEGF) and various chemokines [100,111]. Moreover, the trigger of an acute or chronic inflammation response leads to a further infiltration in the ischemic tissue by pro-inflammatory mediators, by way of the intercellular space between the vascular endothelial cells (i.e., BRB), resulting in a rapid microglial/monocytic response and gliosis [111]. 

The BRB is a functional neurovascular structure composed of two barriers: the retinal vascular endothelium (inner part) and the retinal pigment epithelium (outer part). Under normal conditions, the BRB, similar to the blood brain barrier (BBB), prevents extracellular fluid accumulation through specific tight cell-cell junctions [112] and maintains the retina as an immune-privileged site, thus stopping the passage of both systemic immune and inflammatory components [113]. Indeed, local immune suppression in the eye is achieved through the blood-aqueous, the BRB, as well as the local production of immunosuppressive cytokines and neuropeptides, which promptly eliminate, by apoptosis, any immune cells which have entered the retina in response to an infection [114]. 

However, in glaucoma, the presence of either foreign/auto- antigens or an increased IOP lead to neuroinflammation by way of both toll-like receptor (TLR) and tumor necrosis factor receptor (TNFR) signaling-pathways, the glial NF-ĸB activation and the assembly of inflammasome [93,115,116,117,118]. 

The resulting increase in inflammatory mediators (i.e., the Complement system) and cytokine secretion (i.e., TNFα) are responsible for the reduced BRB efficacy. The edema formation, in turn, disrupts the functioning of surrounding cells by compression [119]. Moreover, the increase in the Major Histocompatibility Complex Class I and II by VEGF and the Heat Shock Proteins leads to the activation of an adaptive immune response and, consequently, the disruption of the BRB [111,120]. 

An impairment in both BBB and BRB may also be found in aging, resulting in an inflammatory or immune response. In fact, despite such impairments not being observed in all aging conditions, during age-related neurodegeneration, including glaucoma, there is sufficient evidence that T lymphocyte infiltration causes microglia activation and RGC degeneration [121]. 

Previous studies have shown differences in T-cell subsets between blood samples of glaucomatous patients and healthy subjects [10,122], leading to the assumption that T- lymphocytes could be involved in the initiation and progression of some glaucomatous lesions [10]. In fact, the lymphokine pattern relating to various T-lymphocyte subsets plays a crucial role in worsening or switching off the immune response (i.e., Th1 secrete IFNγ and IL2, Th2 secrete IL4 and IL10, T suppressor/cytotoxic 1 secrete IFNγ and T suppressor/cytotoxic 2 secrete IL5 and IL10).

**Figure 2:**
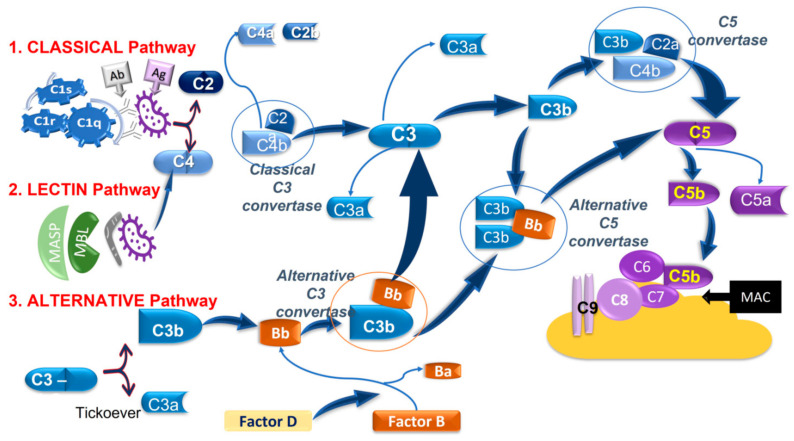
The Complement pathway: three pathways activate the Complement cascade, and they converge within this.

At present, new experimental evidence has clearly shown the role played by glial cells, together with dendritic cells and invading monocytes in triggering the adaptive immunity during glaucomatous neuroinflammation [123,124]. Such conditions, favoring the interaction between glia and T-cells, boost the immune response and lead to an immune-degenerative response [10,122]. Furthermore, it would seem that some proteins belonging to the Complement system, including C1q, C3 and C4, are mainly up-regulated at both the optic nerve head (ONH) and the inner plexiform layer in the early stages of glaucoma degeneration [5,125]. In particular, Hubens et al. [126] have shown that the ratio C3a:C3 was significantly increased in both the serum and the AH of only the progressive POAG patients compared to the stable POAG patients. Therefore, the rising levels of anaphylatoxin C3a is related to the increased cleavage of C3 and the consequent release of C3b, which then binds to the C3 convertase (C4bC2a), forming a new enzymatic complex, namely C5 convertase. In turn, the C5 convertase cleaves C5 to bioactive fragments C5a (anaphylatoxin) and C5b, with the latter recruiting the last complement components C6, C7, C8, and C9 to form the membrane-attack-complex (MAC) ring, leading to cell lysis (Figure 2). This effect provokes RGC damage exacerbation at a retinal tissue level [127,128]. The increase in Complement activation has been linked to reduced RGC survival both in humans and in animal glaucoma models, suggesting its crucial role in glaucoma progression [19].

(1) The classical pathwayis activated by the interaction between the antibody-antigen (Ab-Ag, respectively) and C1-complex, which consists of C1q, C1r, and C1s. This interaction leads to the cleavage of C4 and C2 and the complex C4bC2a to form the C3 convertase which cleaves C3 into C3b and C3a. Then C3b associates with C4bC2a to give rise to the C5 convertase which, in turn, cleaves C5 in C5a and C5b.
  C3a and C5a are anaphylatoxins, acting as vasoactive and chemotactic factors, while C3b is an opsonin inducing phagocytosis. C5b interacts and activates other complement components, namely C6, C7, C8, and C9 to form the membrane-attack-complex (MAC), which lyses targeted surfaces.
(2) The lectinpathwayis activated by the mannose-binding lectin (MBL) recognition of pathogenic carbohydrate motifs. The complex MBL-associated serine protease (MASP) cleaves C2 and C4 and generates the C3 convertase which then merge at the subsequent step of the classical pathway.
(3) The alternativepathwaystarts from the spontaneous hydrolysis of C3 to the C3b analog, C3(H_2_O), which, in the presence of Factors B and D, forms an alternative C3 convertase, namely C3(H_2_O)Bb, which converts C3 into C3b and C3a, acting as the C3 convertase of the classical and lectin pathways. The alternative pathway can also contribute to forming a C5 convertase (C3bBbC3b) merging at C5 of the classical and lectin pathways [129,130].

## 3. Neuroprotection

Retinal damage in glaucoma is due to the gradual degenerative process that results in a worsening of the retina function and the impairment of the visual microenvironment [131]. Indeed, neuro-imaging technologies showed that POAG patients’ eyes display abnormalities, including the death of a significant number of RGCs and the loss of axons in the optic nerve [132]. 

Although IOP management with surgery or drugs is the main clinical approach, the outcome is not always satisfactory. Generally, this is due to the different and complex mechanisms by which RGCs die, such as mitochondrial dysfunction, oxidative stress, NTdeficits, glutamate excitotoxicity, acute/chronic ischemia, inflammation, and glial activation [133].

Therefore, in addition to well-known hypotensive drugs, either new local or systemic drugs with neuroprotective mechanisms, are needed in order to prevent the progression of visual deficits. The term “neuroprotection” refers to a therapeutic paradigm which is able to prevent glaucomatous neurodegeneration, or at least to slow down the progression of the RGC death [131]. Indeed, the possibility of counteracting some of the different complex pathways underlying the apoptotic cascade could represent the key to RGC protection [134,135].

In general, also under para-physiological conditions, RGCs may suffer damage, such as oxygen deficiency, leading to an impairment in their mitochondrial function, or to a condition of oxidative stress. Therefore, these risks are counterbalanced by certain defense systems, which, depending on the context of the retinal microenvironment, instead of limiting the damage, may actually contribute to glial cell activation and, as a consequence, lead to the progressive deterioration of retinal function [135,136].

In this regard, the more promising neuroprotective approaches are based on the increase of antioxidant activities given that oxidative stress, like mitochondrial dysfunction, can be considered common denominators of all glaucoma forms [137]. In fact, the integrity of RGCs can be affected by an imbalance between ROS production and the inefficiency of antioxidant defenses, allowing mitochondrial dysfunction to play both a direct and indirect role in RGC apoptosis [138,139,140,141]. Thus, several therapeutic approaches have recently been focused on the protection of mitochondrial functions, as well as on the increasing of antioxidant systems, in order to protect the RGCs from damage and from the consequent risk of apoptosis activation. Since these approaches contribute to the maintenance of mitochondrial integrity, they can be considered a useful tool in the neuroprotection field [142].

### 3.1. Müller Glia and Neuroprotection

Müller glial cells (MGCs), which together with astrocytes form a part of retinal macroglia, play an important role in maintaining retinal homeostasis by supervising the ionic exchange and the response to neurotrophic molecules [143,144]. Moreover, MGCs exert a real neuroprotective effect by releasing NTs and carrying out antioxidant functions, as well as regulating neuronal signaling by recycling neurotransmitters [145]. 

The function of NTs has already been discussed in the previous section, but it is important to highlight the role of MGCs in the regulation of such factors, which are selectively produced in response to surrounding stimuli. MGCs control the uptake and clearance of glutamate, preventing neurotoxicity. If an excess of this neurotransmitter occurs in the extracellular environment, MGCs up-regulate BDNF, NGF, NT-3, NT4 and GDNF levels to avoid glutamate toxicity [146]. Furthermore, the release of neurotrophins by MGCs occurs also in the presence of other NTs, inflammatory cytokines and activated macroglia [65,147,148]. 

MGCs control the physiological antioxidant response through the expression of high levels of both glutathione (GSH) and glutamate transporters [149,150] and, during oxidative stress conditions, they also regulate the nuclear factor erythroid 2-related factor 2 (NRF2) expression [151,152].

In several in vivo and in vitro studies, it has been demonstrated that kidney, liver, and brain cells express a cystine-glutamate antiporter, which facilitates the exchange between cystine and glutamate, promoting GSH synthesis as a consequence. This exchange is crucial in the retina microenvironment, where, under ischemia or other redox imbalances, the extracellular concentration of glutamate is high, and therefore GSH levels have to be increased in order to counteract the oxidative stress conditions [153,154,155,156,157].

Lastly, another important function of MGCs is the direct control of neuronal-signaling by recycling of both excitatory (i.e., glutamate) and inhibitory (i.e., GABA) neurotransmitters [158,159,160]. Under pathological conditions, such as ischemia, the loss of extracellular glutamate/GABA regulation leads to the loss of MGC neuroprotective effects.

MGCs, due to their intrinsic role in counteracting stress damage, are also involved in inflammation and cell repair/survival processes. [145,161,162,163,164,165]. Indeed, under pathological processes, the establishing of a reactive gliosis results in the MGC release of vasoactive molecules and pro-inflammatory factors [166]. Therefore, during aging, cell injury and oxidative stress, an impairment of GSH content and an increase in TNFα levels occur, which, in turn, lead to other damaging conditions, such as lipid-peroxidation cascade, ferroptosis, autophagia, senescence and death pathway (i.e., apoptosis or necrosis) [167], exacerbating neuronal dysfunction. Although these outcomes are known to be strictly correlated with one another, their prevalence would seem to be dependent on the particular cell model on which they are analyzed [168].

### 3.2. Neuroprotective Agents in Glaucoma Management

#### 3.2.1. Antioxidants

To maintain redox homeostasis, multiple therapeutic targets, on which endogenous and exogenous neuroprotective molecules may act, have been proposed. 

For instance, Vitamin E (i.e., α-tocopherol, γ-tocopherol and TPGS) has been shown to reduce the edema formation during ischemia-reperfusion injury [169]. 

Coenzyme Q10 (CoQ10), which is an important component of the mitochondrial respiratory chain, when administered as a supplementary therapy slows or reverses pathological conditions, including ischemia and neurodegeneration and also protects against glutamate excitotoxicity [170].

Moreover, the concomitant topical administration of Vitamin E and CoQ10 reduce both retinal damage and RGC loss, thus preventing mitochondrial permeability transition pore formation and the release of cytochrome c [171]. 

Furthermore, Nebbioso et al. [172] demonstrated that the treatment with alpha-lipoic acid and superoxide dismutase exerts anti-apoptotic effects and protects against oxidative stressin glaucoma animal models. In addition, intravitreal pre-treatment with recombinant adeno-associated virus expressing SOD2 inhibits RGC death, despite IOP elevation [173]. 

Plant foods, which are the so-called exogenous antioxidant molecules, are rich antioxidant sources. In this regard, the relation between their dietary intake and glaucoma [174,175,176] is currently being assessed. In glaucoma patients, the dietary intake of Vitamin A and C showed soothing effects on the disease progression [177]. Moreover, quercitin supplementation has evidenced a protection on the mitochondrial function in a rat chronic ocular hypertension model and in primary-cultured RGCs exposed to hypoxia [178] whereas Ginkgo Biloba extract and anthocyaninsimprove the general visual function of patients affected by normal tension glaucoma [179]. Lyciumbarbarum extracts, in rat PC12 neuronal cells, showed a neuroprotective action towards glaucoma onset by modulating the expression of extracellular matrix proteins and polysaccharides, preserving the RGCs from oxidative stress-induced apoptosis [180,181].

Carotenoids also evidenced neuroprotective effects, exerting remarkable anti-inflammatory and antioxidant action in the retina. In particular, among carotenoids, lutein- and axanthin-derived molecules, have been studied in several in-vitro and clinical studies [182,183,184,185,186,187,188,189,190]. 

However, despite the large number of available antioxidants, the limits of their therapeutic use are represented by a failure to reach the relevant sites of free radical generation (e.g., mitochondria). The use of specific mitochondrial-targeted peptides with targeted delivery antioxidants to the inner mitochondrial membrane as in the case of Szeto-Schiller peptide 31 (SS-31), can at least partly overcome this problem [191,192,193]. In fact, SS-31, in reducing ROS-mitochondria levels, exerts neuroprotective effects directly on RGCs and antioxidant effects on the retina [194]. 

#### 3.2.2. Neurotrophic Factors (NTFs)

In addition to antioxidant molecules, also neurotrophic factors are the focus of basic research and clinical studies due to the fact that a lack of neurotrophic factors contribute to the impairment of the axonal and synaptic functions (see the previous section) [5,135,136,195]. 

Although NTF-signaling pathways are subjected to a complex regulation, that does not always favor cell survival, different techniques for restoring NTFs have been developed. 

It is now known that in HTG, the increase in IOP leads to reduction of NGF content in cerebrospinal fluid and lateral geniculate nucleusas well as a reduced expression of its receptorsin brain visual centers [196,197,198]. Lower NGF levels or a lack thereof are also found in age-related neurodegenerative diseases (e.g., AD neurodegeneration) [199]. Therefore, chronic NGF administration has been proposed to counteract both AD and glaucoma neurodegenerations. In the particular case of glaucoma, it has been shown, both in rat glaucoma models and in three patients with advanced glaucoma, that repeated topical administration of exogenous NGF prevented RGC apoptosis or, at least, resulted in a long-term improvement in their eyesight. 

However, glaucoma degeneration results in generalized NT deprivation, which, together with additional obstruction to retrograde transport, contributes to disease progression. In this regard, further studies have been aimed at restoring the loss of other NTs, such as BDNF and CNTF. Indeed, repeated intravitreal injections of purified BDNF or CNTF exert neuroprotective effects by reducing the amount of RGC loss, even though BDNF was more effective than CNTF since, after four injections, it was seen to increase the RGC survival by about 73–83% compared to the uninjured controls [56,200,201]. Nevertheless, given the issue related to their sustained delivery, this approach has not found a feasible clinical translation for treating glaucoma. In this context, the introduction of slow-release devices, such as microspheres of poly-DL-lactide-coglycolide (PLGA) infused with NTFs, allow for a sustained NTF release after microsphere degradation, even though repeated treatments are necessary. In fact, with this approach, after repeated injections of microspheres, infused with GDNF, the RGC survival of DBA/2J mice was increased up to nine months [202]. Moreover, gene therapy, using a viral vector (e.g., adenovirus), could improve RGC degeneration long term through a transient up-regulation of specific NTFs [57,203]. 

Even though several pre-clinical studies have demonstrated that NTF supplementation could attenuate the RGC loss, it is necessary to better understand the NTF-signaling pathways, as well as improving the delivery strategies with which NTFs are administered. 

#### 3.2.3. Novel Neuroprotective Agents

Cytidine 5′-diphosphocholine, better known as Citicoline, already finds its therapeutic use in systemic neurodegenerations, such as AD, PD and ischemia. In fact, citicoline, since its chemical structure resembles a precursor of phosphatidylcholine, has several action mechanisms, including phospholipid homeostasis, redox homeostasis, mitochondrial dynamics, cholinergic and dopaminergic neurotransmission [204,205,206]. Therefore, given the similarities between the neurodegeneration pattern of glaucoma and other neurodegenerative diseases (e.g., AD), citicoline has become increasingly used also in glaucoma studies showing its ability to stabilize the plasma membrane of RGC axons and to counteract glutamate excitotoxicity [207], as well as to control oxidative stress. 

Synthetic Bile Salts (SBS), i.e., Ursodeoxycholic acid and tauroursodeoxycholic acid, are used for hepatic and cardiovascular diseases, and for neurological signaling both in physiological and pathological conditions [208,209,210,211]. Moreover, the biological potential of SBS also includes a role in stem-cell differentiation, in promoting the anti-apoptotic pathway, together with Unfolded Protein Response (UPR) signaling, as well as antioxidant activities [212,213,214,215,216,217].

Therefore, SBSs should also be considered as neuroprotective agents for their involvement in anti-inflammatory actions, acting as inhibitors of NF-κB-signaling in glial cells and, at the same time, as promoters of the TGFβ-cascade pathway [218,219]. 

Recently, further innovative retinal neuroprotective approaches, have been targeted to fight the numerous molecular events related to retinal damage. Among these, the antagonist of endothelin receptors [220] and nitric oxide donors [221] have been studied. In addition, also progesterone has been proposed since it plays multiple roles in neuroprotection in terms of axonal transport regulation, increase of NTs, modulation of pro-inflammatory processes, and reduction of GABA-induced neurotoxic effects [222,223,224,225,226,227,228].

Furthermore, cannabinoids have shown neuroprotective potential in various neurological disorders by modulating oxidative stress, the inflammatory response, and glutamate-induced excitotoxicity. The neuroprotective action is mediated by their capability to activate the PPARα signaling, which inhibits the NF-ĸB pathway, thus increasing the transcription of anti-inflammatory cytokines, as well as GDNF and BDNF neurotrophins [229,230,231,232,233,234,235].

**Figure 3:**
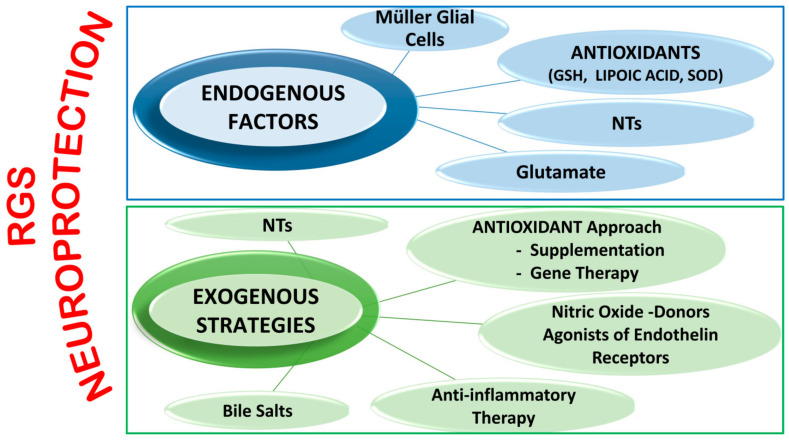
Neuroprotective mechanisms for preventing and slowing down RGC diseases.

It is important to underline that several other innovative neuroprotective approaches are currently being assessed (Figure 3). However, since almost all studies are based on animal models, attempts to perform clinical trials has resulted in many difficulties due to the results not necessarily reproducing those obtained in animal glaucoma models, which in themselves differ from one species to another. Moreover, some of these potential drugs have shown not only instability and difficulty in their delivery to the retina, but also several adverse outcomes [135,236]. 

Endogenous factors include Müller Glial Cells functions, antioxidant systems, NTsand glutamate.

Exogenous neuroprotective approaches includeNTsand antioxidant supplementation or gene therapy, molecular donors of nitric oxide, agonists of endothelin receptors, drugs for counteracting inflammation pathway and bile salts. 

## 4. Conclusions

Although the first-line treatment for patients with glaucoma is lowering the IOP, it should be taken into consideration that glaucoma manifests various and important underlying molecular changes, which, in a certain way, lead to RGC death. 

Therefore, it is crucial to increasingly broaden our knowledge of the initial conditions that bring about glaucoma, such as those reported in this present review, to develop new therapeutic approaches.
